# An Examination of Misconceptions and Their Impact on Cervical Cancer Prevention Practices among Sub-Saharan African and Middle Eastern Refugees

**DOI:** 10.1089/heq.2020.0125

**Published:** 2021-06-01

**Authors:** Selemawit Ghebrendrias, Sarah Pfeil, Bonnie Crouthamel, Morgen Chalmiers, Gennifer Kully, Sheila Mody

**Affiliations:** ^1^ Division of Family Planning, Department of Obstetrics, Gynecology and Reproductive Sciences, University of California, San Diego, San Diego, California, USA.; ^2^ Department of Obstetrics and Gynecology, University of California, San Diego, San Diego, California, USA.; ^3^ Department of Obstetrics and Gynecology, University of California, Davis, Davis, California, USA.

**Keywords:** cervical cancer screening, HPV vaccination, pap test, refugee women

## Abstract

**Objective:** The purpose of the study was to understand cervical cancer screening and prevention practices of refugee women in San Diego, California and identify desired components of a cervical cancer screening toolkit.

**Methods:** We conducted a qualitative study utilizing semi-structured focus groups and identified common themes via grounded theory analysis.

**Results:** There were 53 female refugee participants from Sub-Saharan Africa and the Middle East. Over half of all women surveyed expressed a fear of pelvic exams and loss of modesty as barriers to seeking gynecologic care, with nearly 34% avoiding routine pap tests. Of the 18 participants who were asked if they were aware of the Human Papilloma Virus (HPV) vaccination, only one had heard of the vaccine and none had received it for themselves or their children. Over 60% of participants were interested in educational materials surrounding HPV and pap tests.

**Conclusion:** There is a significant lack of knowledge regarding cervical cancer screening and HPV vaccination among refugee women in San Diego, California. Refugee women in this study were interested in multi-modal educational materials as part of a cervical cancer screening toolkit.

## Introduction

Globally, rates of cervical cancer are increasing, predominately in low- and middle-income countries.^1^ Over 100,000 women in Sub-Saharan Africa are diagnosed annually, with over 60% dying from the disease without treatment.^2^ Persistent infection with high-risk, oncogenic human papillomavirus (HPV) leads to the development of precancerous lesions that subsequently progress to invasive cancer without intervention.^3^ There are multiple ways to screen for cervical cancer. In resource-rich nations with access to pap tests, a pelvic examination is performed with a speculum and cervical cells are collected to assess cytology and for the presence of HPV.^4^ In low-income countries where routine screening is limited, there is a significant underestimation of HPV prevalence.^5^ Cervical cancer screening and HPV vaccination for women migrating from these high-risk areas of the world are of utmost importance.^6^

On arrival to the United States, refugees, and African immigrants in particular, are less likely to utilize cervical cancer prevention resources.^7^ This is true even when compared to traditionally underserved communities, including U.S.-born Black women, who have three times the rate of cervical cancer screening relative to African immigrants.^8^ While we know socioeconomic limitations and cultural barriers can impede access to routine gynecologic care, there are few studies assessing the extent of this disparity for African and Middle Eastern refugee women.^9^

In focus groups with migrant and refugee women in Boston, women reported avoiding care when asymptomatic and participating in gynecologic examinations and pap tests only when pregnant due, in part, to the perceived threat to their modesty.^10^ In addition, the ways in which refugees navigate the health care system are more complex than those of immigrants who have not had the defining experience of life in a war-torn community or refugee camp where gynecologic care may have been provided under coercion.^11^ Over 10,000 refugees were resettled in San Diego between 2014 and 2019 alone, as the county has one of the highest resettlement rates in the nation.^12^ Participation in preventative health care is impacted by the breadth of their cultural and religious characteristics and experiences, which for these women are often rooted in Christian and Islamic faiths.^13–15^

In an effort to tackle this issue, the Center for Disease Control and Prevention reviewed various interventions, from faith-based community partnerships and self-collected sampling, to the use of health promoters and educational materials.^16^ Currently available resources are sparse and designed without considering women who are unable to read in either English or their native language.^17–22^ The aim of this study is to understand the current cervical cancer screening practices and perceptions of both pap tests and HPV vaccination among refugees in San Diego, California, and to identify desired components of a culturally centered screening education toolkit.

## Methods

### Study design

This qualitative study was conducted in San Diego, California, with semistructured focus groups. From these focus groups, common themes were identified through grounded theory analysis. Unlike alternative methods of qualitative analysis, such as ethnography or phenomenology, grounded theory provides a systematic approach for the discovery of collective patterns within the data.^23,24^ This study was part of a larger project exploring the experiences of refugee women seeking reproductive health care. Eligibility criteria included self-identifying as a refugee woman of reproductive age, who then agreed to participate in the focus groups. This study was approved by the University of California San Diego Institutional Review Board.

### Recruitment

Participants were recruited by refugee community partners utilizing flyers translated in their respective languages, telephone communications, and in-person discussions. Community leaders associated with the United Women of East Africa Support Team (UWEAST), which provides resources for refugee women from the Middle East and Sub-Saharan Africa, facilitated the outreach to women actively involved in UWEAST events and programs.

### Focus groups

A total of 6 semistructured focus groups were conducted by interpreters recruited through UWEAST, with 6 to 12 participants per 2-h session. The focus groups were completed between February 2019 and March 2020. Participants were unique to each group and were compensated with $50 dollar gift cards. An interview guide was utilized to assist in conversation (Appendix A1). Audio recording was performed with the permission of participants. Written notes were also taken by an English-speaking co-investigator present during the session, who documented the translation provided by the in-person interpreters. Languages requiring interpretation included Somali, Arabic, and Swahili. With the exception of focus group 2, which consisted of only Somalian women, each focus group included multiple ethnicities with non-English speakers and bilingual women.

### Analysis

**FIG. 1.**
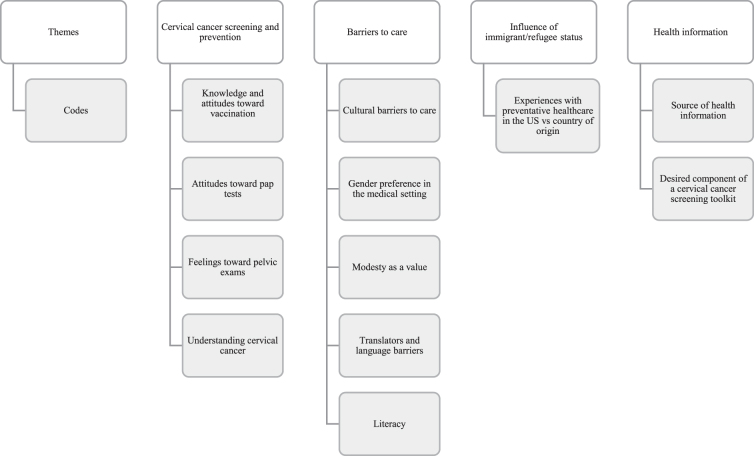
Diagram of themes.

Using these transcripts and a grounded theory approach, two coders generated a mutually agreed upon set of themes from which a framework was then developed to facilitate coding the transcripts (Fig. 1). Dedoose software was employed to create the codebook utilized by the coders, which went through two cycles of review.^25^

## Results

### Participants

A total of 53 women participated in this study. Participants were from Sudan, Somalia, Kenya, Ethiopia, Eritrea, Congo, Uganda, Syria, Iraq, Egypt, and Morocco. Women ranged in age from 20 to 50 years (Table 1). Across all focus groups, quantitative data for the number of women who participated in the discussion of each theme and provided statements utilized in the development of the codebook are listed in Table 2.

**Table 1.** Demographic Characteristics of Participants

	Focus group						
Participant characteristics	Total	FG 1	FG 2	FG 3	FG 4	FG 5	FG 6
Ethnic origin							
Sudan	6	3	—	—	3	—	—
Somalia	8	—	6	—	2	—	—
Kenya	2	—	—	2	—	—	—
Ethiopia	3	—	—	—	3	—	—
Eritrea	3	2	—	—	1	—	—
Congo	8	—	—	8	—	—	—
Syria	16	—	—	—	—	8	8
Iraq	2	—	—	—	—	1	1
Egypt	2	—	—	—	2	—	—
Uganda	2	—	—	2	—	—	—
Morocco	1	1	—	—	—	—	—
Age range		31–48^a^	30–50	20–48	33–43^b^	20–45	20–45
Total participants	53	6	6	12	11	9	9

^a^ A total of two participants declined to report age.

^b^ A total of eight participants declined to report age.

**Table 2.** Quantitative Summary of the Number of Women Who Participated in the Discussion of Each Theme and Provided Statements Utilized in the Development of the Codebook

Themes with summarized findings	Total number of participants for all focus groups combined (*N*=53)	Total number of participants present for discussion of this specific topic^a^	Total number of responses received in the form of a statement or gesture of agreement^b^	Percent of contributing participants in agreement with the statement (%)	Percent of participants across all focus groups in agreement with the statement (%)
Cervical cancer screening and prevention					
No previous knowledge regarding an HPV vaccine		18	17	94.4	32
Negative attitudes toward pap tests		47	18	38.2	33.9
The cause and/or prevalence of cervical cancer is unknown		27	15	55.5	28.3
Avoidance of and/or fear of pelvic examinations		20	7	35	13.2
Barriers to care					
Cultural differences		47	30	63.8	56.6
Loss of modesty		22	10	45.5	18.9
Lack of translators and language barriers		53	20	37.7	37.7
Influence of immigrant/refugee status					
Access to care limited by challenges navigating the U.S. health care system and working with U.S. health care providers		53	32	60.3	60.3
Health information					
Expressed interest in more health education material and/or specifically a cervical cancer screening toolkit		44	33	75	62.3

^a^ All the interview questions referenced in Appendix A were not asked of every participant directly (i.e., participants in focus groups 1–3 were not asked specifically about the HPV vaccine or the cause/prevalence of cervical cancer, and so they were not included in those “present” for the discussion of that particular topic).

^b^ This total represents the number of individual statements referenced for each code, one statement may be inclusive of multiple topics and the totals reflect this.

HPV, human papillomavirus.

### Focus group themes

#### Theme 1: Cervical cancer screening and prevention

##### Lack of knowledge about the HPV vaccine

Of the 18 women asked specifically about the HPV vaccine, 17 did not know there was an immunization for the prevention of cervical cancer. In addition, all 18 reported that neither they nor their children had received the HPV vaccination. There was also a perception that vaccination was only for females, evidenced by a participant who stated, “You're saying cervical cancer, but how does that have to do with boys.” Despite this lack of exposure, participants expressed interest, as one stated, “All I need you to do is talk to me about it” and another, “I will have to go get the vaccine.” Past experiences with side effects following vaccination upon arrival in the United States deterred some participants from seeking out any future vaccination.

##### Misconceptions surrounding the cause and prevalence of cervical cancer

When women were asked to expound on their understanding of cervical cancer, 55% of participants were not aware of it before the focus group and some perceived it to be a new threat they had not encountered in their native country. One participant stated, “People have more fear about [cervical] cancer because it's something new…it's kind of a little bit shock,” which was a sentiment shared by another, “Back home I have never heard of someone who has [cervical] cancer” and another stated, “So common in America people are getting a lot of different cancer.” Women voiced a variety of suspected causes of cervical cancer, as one woman described aspects of life in America putting her at risk, “Sometimes we can't trust the food, medicine…even the equipment the doctors use.” Multiple participants referenced cancer as a potential side effect of birth control, as one stated, “A lot of times women…they're afraid of side effect like cancer” and another mentioned her sister's concerns, “She believes that if she take birth control, you'll bring cancer.”

##### Negative perceptions of pelvic examinations and pap tests

Across multiple focus groups, women conveyed negative associations with pelvic examinations and pap tests, with 35% reporting an avoidance of pelvic examinations primarily due to fear. As one woman stated, “I prefer not to go for pap smear” and another, “When the doctor say that on that day you're going to have a pap smear, I was so terrified. I don't like it.” The sentiments of multiple participants were summarized by an interpreter who relayed, “in their culture or in their country, they don't do it. It's culture for them to not do it. So here they refuse to do it” and, “the reason they don't like it, the pap smear, they are shy to open the legs or put the things, the pain, the pressure.” Many participants expressed a fear of metal speculums causing local trauma, pain, or pressure with insertion, as one recounted, “When I saw the material with metal, I was scared and my heart started beating loud” and another in reference to the lack of previous exposure to speculums, “When I was in Egypt, they touch the woman with hands. She doesn't feel anything and she's quiet.” There was also uncertainty regarding the interpretation of pap test results which was detailed as a limiting factor to testing, “One time I did, and they tell me… they pretend I have cancer.” Despite this fear and confusion associated with pap tests, multiple participants reported a willingness to engage in screening at the request of their physician and with encouragement of family and friends.

#### Theme 2: Barriers to care

##### Modesty as a barrier to gynecologic exams

The fear and discomfort with physical exposure was a stronger core value than participating in gynecologic care for many participants as 45% reported avoiding care due to this concern. This was a consistent theme across multiple focus groups, as one woman highlighted, “I don't feel comfortable with that…I cannot open my legs,” another recounted her experience, “…just scared to open my legs” and another questioned, “…why should I have to go? To open my private parts?.”

##### Perceived religious stereotypes and misinterpreted traditional gestures as a cultural barrier

Some Muslim women recalled feeling as though their physician was fearful or reticent in their interactions because of their religious garb. Referring to doctors, one participant stated, “They see us as ‘You Muslim, you covered’ and they scared…they scared to understand you to ask you more questions. Sometimes they try to shake your hand and say, ‘I'm sorry, I'm sorry’.” Traditional gestures of respect such as avoidance of eye contact were misinterpreted by U.S. health care providers to be a lack of engagement in health care conversations. Multiple participants referenced this behavior in particular. One stated, “That eye to eye contact, when we respect people we don't look them like this,” and another reported, “Africa culture, when you talk to someone, especially us, woman, we have to respectful talk to us, we have to put our eyes down” and another stated, “People maybe they might think, like, you bad.”

##### Discussions of gynecologic issues are considered to be a cultural taboo

Women described the challenges of having an open dialog with friends and family as a reflection of their cultural norms. One participant stated, “Everybody in Africa are secretive” and another noted, “When we come from Africa, like, there's a lot hidden inside of us, but we can't say it, because of the culture.” One woman stated clearly, “I don't think I comfortable to talk about it or to show our body.” Despite these cultural restraints, some participants expressed their desire for transparency and vulnerability. They did, however, feel that they lacked the tools to do so with their daughters, as one stated, “Sometimes I want tell her something but I don't know how to say” and another reported, “We feel shy we don't talk about it like parents and child like that.”

##### There is a strong preference for female providers

One woman stated, “Absolutely. I would prefer, obviously, someone who is a woman.” In emergency settings, which were perceived to be male dominated, women voiced concerns regarding gynecologic-specific care and reported refusal of high acuity care from male providers. Male physicians were also perceived to be a barrier to pap testing in an outpatient setting. When asked what potential barriers they faced in general, one participant stated, “If there was a male doctor.” Even though participants had a strong preference for female physicians, some reported discomfort discussing gynecologic issues with anyone aside from their husband. In contrast, one participant stated, “It's about hospitality. As long as you smile at them and as long as you're being nice and welcome. They have no problem. Male, female, any race.”

##### There was a desire for translation services and specifically in-person translators to avoid missed emotional cues

Even with the use of interpreters, many participants were concerned about the accuracy of translation, privacy regarding shared health information, and variations in dialects. Across all focus groups 37% reported these limitations impacted their care. Some participants also referenced a desire to have providers from their country of origin, as one woman stated, “So when you see your own people speaking through your own language, you building trust.” Despite this, some reflected on the fact that shared language does not equal shared culture and even with providers fluent in their native language, cultural barriers can limit interactions.

#### Theme 3: Influence of immigrant and more specifically refugee status on access to health care

##### The lack of exposure to preventative health care, such as mammograms and pap tests, in their home country contributed to the perception of preventative health care as a novel idea in the United States

Of the 53 participants, 32 statements were made referencing the U.S. health care system's emphasis on preventative health and the ways U.S. health care providers impact access to care. Some participants did not receive or value preventative gynecologic care, including cervical cancer screening on arrival to the United States. Examples of common sentiments regarding pap tests include, “In my country, we don't use it… We don't have those things,” along with, “Pap smear tests or the mammogram or the breast cancer test, um, it's something honestly I felt it's a big deal over here…where I used to live or where I come from, people go when they feel pain” and, “The doctors office called us and said, pap smears. I said, ‘Why should I have to do, why I don't have a problem. Why do I need pap smears?’.” Some participants reported a dearth of screening while in refugee camps, and on arrival to the United States, their initial exposure to pap tests was in pregnancy. “We don't know it. You think about when you done with babies, you done. Don't go to the doctor.” Another woman stated, “I'm not having a baby, I don't have a husband. Why should I go open my private to the doctor?,” with a similar sentiment shared by another, “She cannot imagine any material go inside, only when she gets delivery. After that she doesn't like anything go in.”

#### Theme 4: Health information

##### Women's health information is obtained from family, friends, and physicians

Many participants reported watching YouTube videos and seeing posts on Facebook and discussing what they learned with their friends. Across multiple focus groups, women reported their children relayed health information they learned in school, “Our kids are like our teachers now.” Physicians were seen as a trustworthy source of information as one stated, “I trust the doctor that if I ask a question, he would sit and explain it to me very well.” After discussing the importance of gynecologic care, one participant expressed her motivation to not only make appointments for her daughters but also for herself as she and the interpreter stated, “I didn't talk about before. Now I know I need to” and “I think we all need to do appointments.”

##### There is a desire for more educational materials

Nearly 75% expressed interest in improved health education materials. Various responses included, “Even though the doctor explains, it would be nice to kind of give them a brochure to talk more information about it so they're able to understand it more,” and another stated, “There are a lot of women who have the same, similar issues, and having a space like this to talk about the problems that they have with feminine health would be good.” For mode of delivery of information requests included, “do it in social media, Facebook and WhatsApp and send for everybody,” “I'm a visual learner, so I think a picture would be nice,” “If you give us pictures it could be helpful” and “video and book.”

## Discussion

In this qualitative study of semistructured focus groups with Sub-Saharan African and Middle Eastern refugee women who have resettled in San Diego, California, perceptions and attitudes toward cervical cancer prevention were explored. In addition, desired components of a cervical cancer screening toolkit were determined.

Significant findings included a lack of knowledge surrounding the prevalence and cause of cervical cancer, the purpose of pap tests, and interpretation of results. Many women described unfamiliarity with routine health screening in general and only saw the value in gynecologic care when they were feeling unwell or were pregnant. This particular point of view was shaped by experiences living in countries and refugee camps where resources for preventative health measures were not available. The vast majority of women had never heard of the HPV vaccination and denied having had their children vaccinated. There was also confusion regarding the recommendation to vaccinate male children. Linguistic barriers were also an important factor as many of these women require translation services when receiving care and have limited literacy in both English and their native language. This was likely one of the leading reasons participants requested visual aids when discussing ideal methods of providing health education.

While culture and religious beliefs influence their access to all forms of health care, it is particularly salient in the context of gynecologic care.^26–29^ In this study, there was substantial fear of pelvic examinations, from the pain associated with speculums to the concern for modesty. Women also detailed a lack of comfort discussing these topics with their family and friends, particularly their daughters, as a result of cultural taboos.

Multiple limitations of this study were identified. While great effort was put into drawing in a diverse group of women, participants were predominately Syrian. The initial focus groups were focused primarily on access to reproductive health care, with only brief discussions of cervical cancer prevention. In an effort to maintain a focus group format and avoid direct interviews or surveys of participants, not every question was asked of every participant, which limited the sample size for specific topics discussed. The transcriptions did not identify participants by country of origin, preventing comparisons between ethnic groups. Finally, recorded statements were not designated in the transcriptions to a specific participant, which may affect the quantitative summaries provided. However, to mitigate this, multiple statements made by the same participant were grouped as a single response so that the number of individual statements would be equivalent to the number of individual participant responses.

Despite these limitations, we found multiple areas of potential intervention, including expanded translation services, language-specific health education materials, and group sessions geared toward pap test and HPV vaccine education.

## Conclusion

This study examined the cervical cancer prevention practices of refugee women from the Middle East and Sub-Saharan Africa living in San Diego, California. Focus groups revealed shared patterns of avoidance of pap tests due to a fear of pelvic examinations and a concern for modesty and lack of knowledge regarding the HPV vaccine. Participants requested culturally centered health education through print and online platforms. From this pool of knowledge, providers can begin to grasp the extent of the disparity and target future efforts toward a multimodal approach to cervical cancer prevention.

## Abbreviations Used

HPV: human papillomavirus
UWEAST: United Women of East Africa Support Team

## Appendix

**Table:** Appendix A1. Focus Group Interview Questions

	Focus group interview questions
Cervical cancer	Do you know where/what the cervix is?
	What do you know about cervical cancer?
Screening practices	What do you know about cervical cancer screening?
	Have you had a pap test?
	What have you heard from women in your community about pap tests and cervical cancer screening?
	Did you ever hear about cervical cancer in your country of origin?
	Did you every have a pap test before coming to the United States?
	Do you get annual examinations, pelvic examinations regularly?
HPV vaccine	What do you know about the HPV vaccine?
	Would you encourage your daughters and sons to have the vaccine?
	What would help you understand why cervical cancer screening is important?
	What would change your mind about the need for pelvic examinations and pap tests?
Health information	Who encourages you to go to the doctor?
	What resources do you use for health information?
	Would you feel comfortable sharing health information with your daughters?
	How often are you online?
Literacy	Can you read or write in your own language?

HPV, human papillomavirus.
